# The emerging role of snoRNAs in human disease

**DOI:** 10.1016/j.gendis.2022.11.018

**Published:** 2022-12-26

**Authors:** Xinhai Zhang, Chenglong Wang, Shujun Xia, Fei Xiao, Jianping Peng, Yuxuan Gao, Fengbin Yu, Chuandong Wang, Xiaodong Chen

**Affiliations:** a Department of Orthopedic Surgery, Xinhua Hospital Affiliated to Shanghai Jiao Tong University School of Medicine (SJTUSM), Shanghai 200092, China; b Ultrasound Department, Rui Jin Hospital Affiliated to Shanghai Jiao Tong University School of Medicine (SJTUSM), Shanghai 200025, China; c Department of Orthopaedic Surgery, Second Hospital of Shanxi Medical University, Taiyuan, Shanxi 030001, China; d Department of Orthopaedics, The 72nd Group Army Hospital of PLA, Huzhou, Zhejiang 313000, China

**Keywords:** Cancer, Genetic disease, Inflammation, Metabolism, Small nucleolar RNAs

## Abstract

Small nucleolar RNAs (snoRNAs) play critical roles in various biological processes. The aberrant expression or depletion of snoRNAs is related to various diseases. In previous research, most of the snoRNAs were categorized as C/D box snoRNAs and H/ACA box snoRNAs, whose typical functions were thought of as regulation of 2′-O-ribose methylation and pseudouridylation of ribosome RNAs, respectively. However, in the past two decades, studies have revealed an increasing number of snoRNAs without specific targets or determined cell functions. These findings indicated that some potential roles of snoRNAs are still unknown. Numerous studies have indicated the correlation of snoRNAs with human diseases. SnoRNAs play various roles in abundant biological processes, and they have great potential in controlling human diseases. This new and rising field could benefit from investigations of the disease pathogenesis, biomarker identification, and the determination of novel therapeutic targets. This review summarized the reports on snoRNAs and the regulation of different diseases in recent years.

## Introduction

In 1968, Weinberg discovered six different low-molecular-weight RNAs with lengths of 100–180 bp in the nucleoplasm and nucleolus of HeLa cell. With the technology restriction at that time, the characterization and localization of these low-molecular-weight RNAs were undefined.^1^ In the 1980s, with the development of trimethylguanosine (TMG) antibodies and nucleolar fibrillary protein antibodies, distinguishing between snoRNAs and snRNAs became possible. Zieve et al first reported the discovery of SNA/U3 in the cell nucleus, which was later known as snoRNA U3 (SNORD3A).^2^^,^^3^

However, at the beginning of the 21st century, with the completion of human genome sequencing and the development of high-throughput sequencing, researchers began to identify the relationships between snoRNAs and several diseases. In 2002, Gallagher et al proposed that the deletion of paternal allele SNORD116/PWCR1/HBII-85 could lead to Prader–Willi syndrome (PWS).^4^ In the same year, four novel snoRNAs were amplified from human total RNA. One of these snoRNAs, named h5sn2, was highly expressed in normal human brain tissue, but its expression decreased significantly in meningiomas, and this finding was the first time that the imbalance of snoRNA expression was affirmed to be associated with cancer.^5^ To date, snoRNAs have become research hotspots in many fields, although their roles in disease control have not been fully verified.

**Figure 1:**
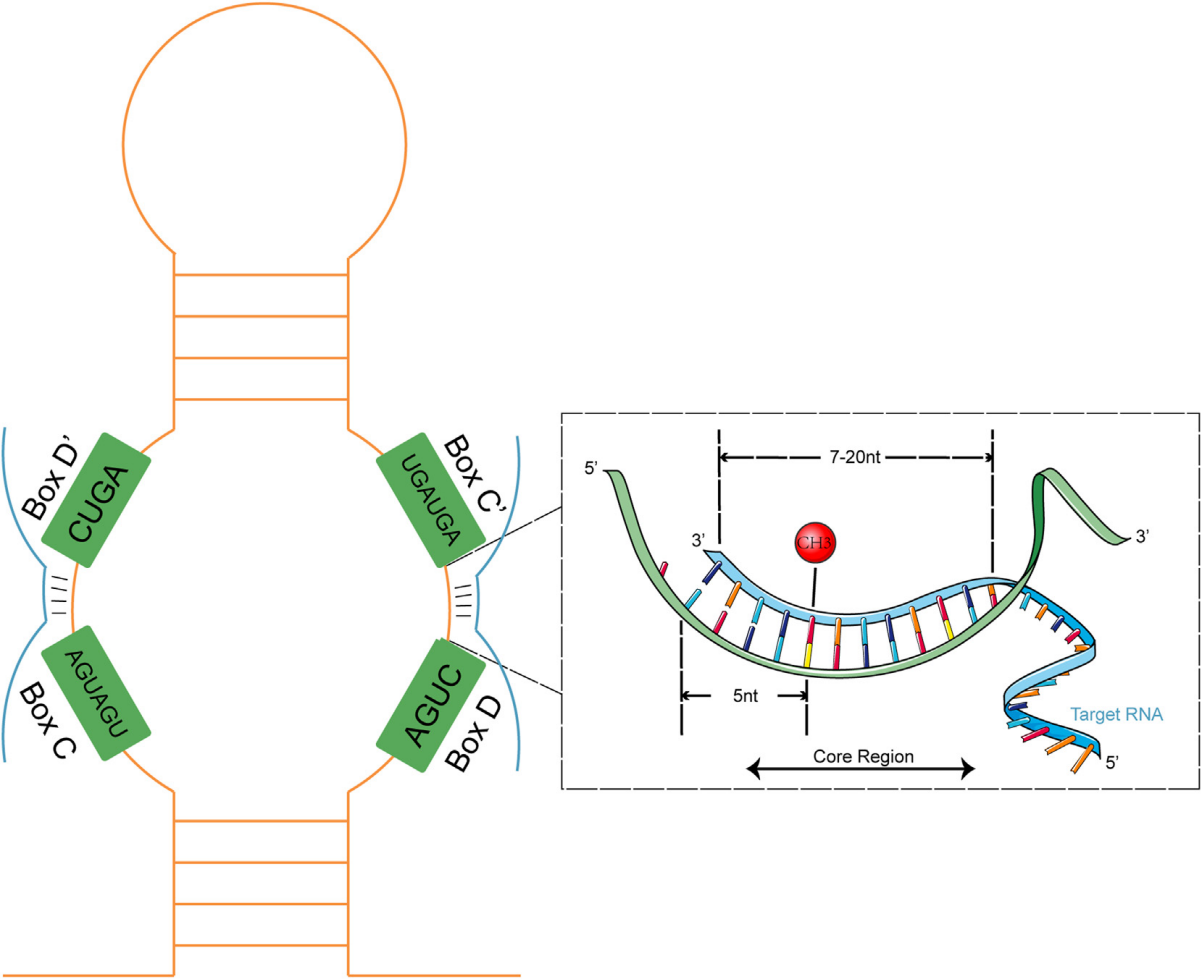
The function of box C/D snoRNA.

**Figure 2:**
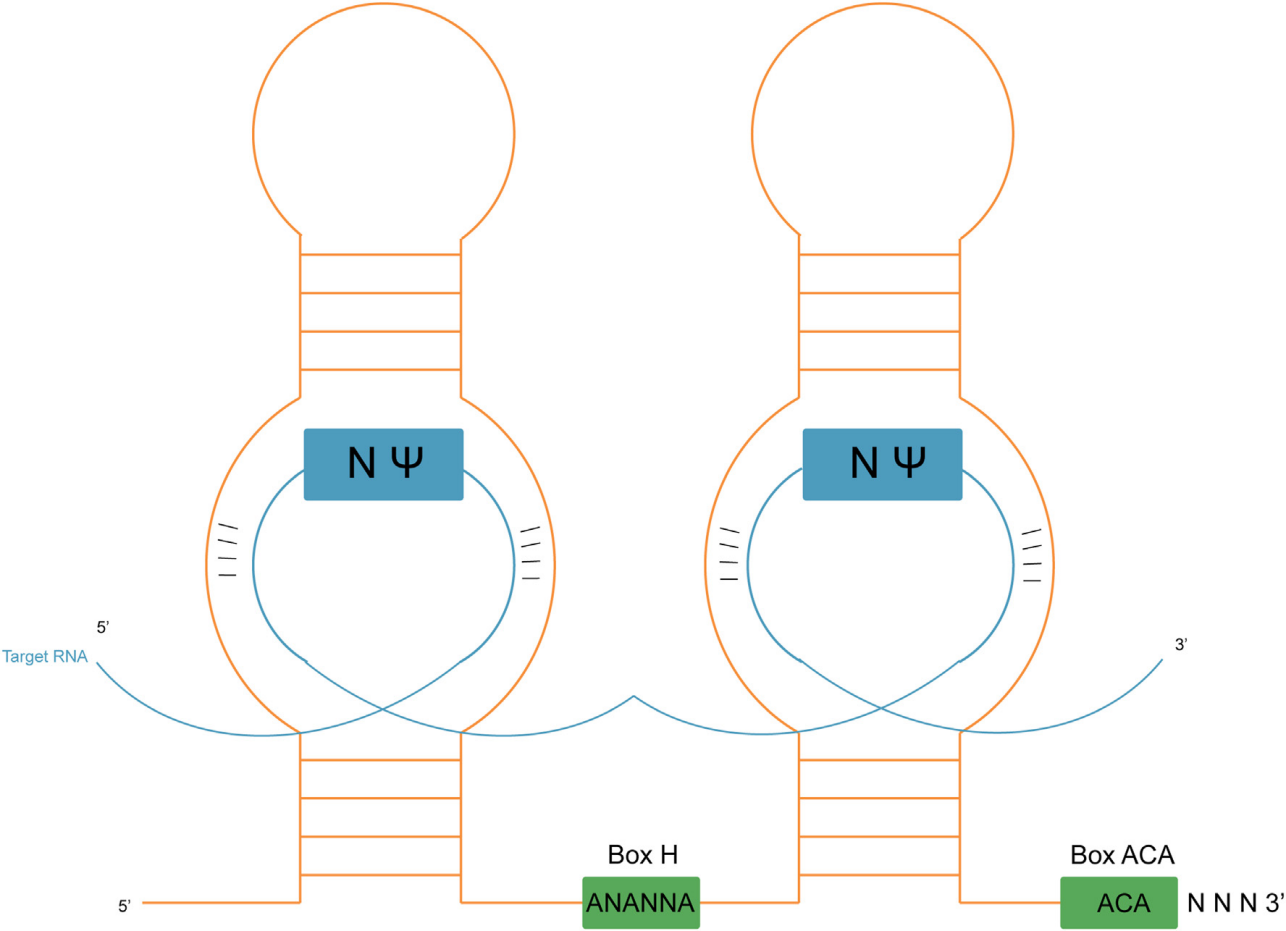
The function of box H/ACA snoRNA.

SnoRNAs are noncoding RNAs (ncRNAs) in the nucleus of eukaryotic cells, with lengths of 60–300 nt. SnoRNAs are either intronic or intergenic, while most of the snoRNAs in humans are intronic which are spliced with debranching and exonucleolytic processing, and the lariat is further processed to a mature snoRNP. Various functions have been identified for snoRNAs. For instance, snoRNAs have been recognized to participate in the maturation process of other types of RNA in cells. SnoRNAs have conserved structural elements, which could be roughly divided into two families, Box C/D snoRNAs, and Box H/ACA snoRNAs.^6^ The structure of typical C/D snoRNAs takes the 5′ end C box (Box C) and 3′ end D box (Box D) as the head and tail, respectively, and the guide region complementary to the methylation target sequence of ribosome RNA (rRNA) is located on the attached upstream region of the D and D′ boxes (Fig. 1). The typical structure of H/ACA snoRNA consists of a connection of two hairpins identifying rRNA pseudouridine target sites with an H box (Box H), with a 3′ end of Box ACA (Box ACA)^7^ (Fig. 2). In addition, different snoRNA families control family size and member abundance by regulating their replication to meet the needs of different individual tissues. Box C/D snoRNA family members are mostly embedded in the same host gene, mainly through *cis* recombination to obtain copy numbers; Box H/ACA family members are often encoded in multiple different host genes, and copy members are obtained through reverse transcription transposition.^8^ In the past 20 years, new snoRNAs have been identified to play unexpected functions. An increasing number of snoRNAs is involved in posttranscriptional regulation, such as rRNA acetylation, splicing mode regulation, mRNA abundance, and translation efficiency regulation.^9^ Therefore, the traditional snoRNA classification could not summarize all the functions of snoRNAs.

## SnoRNA in cancer

### SnoRNAs in hepatocellular carcinoma

In recent years, the roles of snoRNA in hepatocellular carcinoma (HCC) have been the focus of snoRNA research. Many studies have shown that snoRNAs are dysregulated in HCC cells and tissues; for instance, SNORD126 and SNORD105 are significantly up-regulated in HCC tissue, whereas SNORA52 and SNORD31 are significantly down-regulated.^10^, ^11^, ^12^, ^13^, ^14^, ^15^, ^16^, ^17^ A low expression of SNORD113-1 was found in HCC tissue, and the results obtained from a clinical specimen provide evidence that decreased expression of SNORD113-1 contributes to HCC development and progression.^10^ SNORD113-1 inhibited tumor growth through the inactivation of ERK1/2 in the MAPK/ERK signaling pathway and that of Smad2/3 in the TGF-β pathway.^10^ The expression of SNORD126 was enhanced in HCC and promoted carcinogenesis by activating the PI3K-AKT signal transduction pathway via FGFR2.^11^ The same team further proved that SNORD126 regulates FGFR2 expression and activates the PI3K-Akt pathway by binding the hnRNPK protein, resulting in the occurrence of HCC.^11^^,^^12^ In addition to the PI3K/Akt pathway, the Wnt/β-catenin signaling pathway mediates the up-regulation of snoRNA (SCARNA9L and SNORD76) and promotes the occurrence of HCC.^13^^,^^14^ SCARNA9L could inhibit HCC cell apoptosis and induce cell cycle progression, and down-regulating SCARNA9L could significantly inhibit HCC cell proliferation.^13^ By activating key molecules, such as β-catenin, cyclin D1, and c-myc, SCARNA9L may promote the proliferation of HCC cells through the Wnt/β-Catenin pathway.^13^ SNORD76 and SCARNA9L also exhibited similar mechanisms.^14^ The expression of SNORD76 in 66 HCC specimens was quantitatively analyzed, and it was found to be significantly up-regulated in HCC tissues and remarkably associated with worse patient survival.^14^ As verified in cell lines and xenograft nude mice, down-regulating the expression of SNORD76 could induce G0/G1 cell cycle arrest, promote cell apoptosis and the invasiveness of HCC by inducing epithelial–mesenchymal transformation (EMT), and activate the Wnt/β-catenin pathway.^13^^,^^14^

For identification of germline gene copy variation related to HCC, 1583 patients with HCC (patients with chronic HBV coexisting with HCC) and 1540 controls (patients with chronic HBV infection without HCC) from the Chinese population were subjected to a genome-wide association study on the basis of germline copy number variation.^15^ SNORA18L5 on chromosome 15q13.3 promoted the proliferation and tumor growth of HCC cells. Subsequently, SNORA18L5 promoted the maturation of 18S and 28S rRNA by increasing ribosome synthesis. The ribosomal proteins RPL5 and RPL11 were kept in the nucleolus, and their binding to MDM2 was prevented, thus increasing MDM2-mediated p53 proteolysis and cell cycle arrest and finally promoting the occurrence and development of HCC.^15^ Similarly, with p53 as the target, SNORD17 promoted the progression of HCC by forming a positive feedback loop with p53.^16^

Up frameshift protein 1 (Upf1) is famous for its core role in nonsense-mediated mRNA decay. It could eliminate abnormal mRNA carrying a preventive termination codon and prevent the accumulation of nonfunctional or potentially harmful proteins. It is also an important factor in the RNA quality control system. In addition, the expression profile of snoRNAs regulated by Upf1 was detected in HCC cells, indicating that the expression of SNORD52 was up-regulated in HCC and negatively correlated with the expression of Upf1 and clinical prognosis. In subsequent *in vivo* and *in vitro* experiments, SNORD52 was shown to promote the occurrence of HCC by up-regulating CDK1.^17^ By detecting the expression of snoRNAs in 372 HCC tumor tissues and 50 nontumor tissues, nine snoRNAs that could independently predict the prognosis of HCC were identified by bioinformatics analysis; however, these snoRNAs need to be further analyzed in clinical samples.^18^

### SnoRNAs in colorectal carcinoma

Colorectal cancer (CRC) is one of the most common cancers in humans. However, several challenges exist in the treatment of CRC, such as the high recurrence rate, poor prognosis, and serious effects on the post-surgery quality of life. Therefore, developing new and effective diagnostic and treatment strategies for metastatic CRC is still necessary. Research on the regulatory role of snoRNAs in CRC may provide new ideas. By comparing the expression levels of snoRNAs in 35 CRC tissues and healthy mucosal tissues, 41 differentially expressed snoRNAs were found in metastatic and end-stage CRC tissues, of which SNORD12B acquired the largest statistically significant difference.^19^ The correlation analysis of snoRNA with miRNA and mRNA indicated that SNORD12B played a “driver” role in CRC, regulating the expression of other RNAs.^19^ The expression levels of four snoRNAs (SNORD76, SNORD78, SCARNA22, and SNORA42) in 16 CRC samples were detected.^20^ The high expression of SNORA42 was related to the distant metastasis and poor prognosis of CRC in the stage of clinical validation, and it could be used as a biomarker for patients with stage II CRC.^20^ The overexpression of SNORA42 led to an increase in cell proliferation, migration, invasion, tumorigenicity, and anoikis resistance.^20^ Through a public database comparison, Kazuhiro found that SNORA21 was overexpressed in CRC, which is associated with a low survival rate and distant metastasis of patients with CRC. Thus, SNORA21 may be used as a diagnostic and prognostic biomarker for CRC.^21^ Down-regulated SNORA21 expression decreased the carcinogenic potential of CRC cells and inhibited tumor progression in xenograft nude mice.^21^ The expression levels of SNORD44 and GAS5 in tumor samples were lower than those in control samples,^22^ and the expression of SNORD44 was positively correlated with the expression of GAS5 in tumor samples. Subsequently, the researchers constructed an oncolytic adenovirus (SPDD-UG) carrying SNORD44 and GAS5 to overexpress SNORD44 and GAS5. The results showed that SPDD-UG inhibited the growth of CRC and induced cell apoptosis.^22^ Compared with noncancerous tissues, the expression of SNORA15 and SNORA41 in CRC tissues increased, whereas that of SNORD33 decreased. The expression levels of SNORA15, SNORA41, and SNORD33 were up-regulated in the UC and CRC groups. Therefore, SNORA15, SNORA41, and SNORD33 may be involved in the progression from chronic intestinal inflammation to malignant tumors.^23^ SNORA71A, which is located in the chromosome 20q11 region (where the amplification of gene copy number was found in many cancers), had the highest differential multiple. The expression of SNORA71A was significantly correlated with the TNM stage and lymph node metastasis.^24^ Therefore, SNORA71A could be used as a biomarker of CRC.^24^ In addition, the expression of SNORD1C in the serum of CRC patients was significantly enhanced, and its up-regulation was associated with poor tissue differentiation and a high expression of carcinoembryonic antigen. Therefore, serum SNORD1C may be used as a noninvasive tumor biomarker for the diagnosis of CRC.^25^ However, in another study, by analyzing information from the TCGA database, 57 snoRNAs with significant expression differences were found, 50 and seven of which were significantly up-regulated and down-regulated, respectively, in colon cancer. Four prognostically significant snoRNAs (SNORD14E, SNORD67, SNORD12C, and SNORD17) were also found using the uniform Cox expression model.^26^ LncRNA ZFAS1 is a host gene of the SNORD12 family (SNORD12A, SNORD12B, and SNORD12C), which is highly expressed in various tumor tissues.^27^, ^28^, ^29^, ^30^ However, ZFAS1 maintains the methylation function of SNORD12C and SNORD78 by recruiting the SNP protein NOP58 as a scaffolding protein, thereby promoting the proliferation of colon cancer cells.^31^ As SNORD50A has been detected in many cancers, the expression of SNORD50A was reported to decrease during the proliferation of colon cancer cells.^32^ SNORD50A mediates the methylation of 28S rRNA at C2848 and regulates ribosome biogenesis.^32^ SNORD57 and its derived piRNA piR-54265 in the serum of patients with colon cancer could be detected, and they are promising noninvasive biomarkers for colon cancer.^33^, ^34^, ^35^

### SnoRNAs in breast cancer

Research on the regulatory role of snoRNAs in breast cancer could be traced back to 2009. Dong et al found that SNORD50A was down-regulated in breast cancer, and a 2-bp deletion was detected in somatic cell and germ cell lines. In addition, prostate cancer is similar to breast cancer on this point. The overexpression of SNORD50A resulted in colony formation suppression, which indicated that SNORD50A was associated with the occurrence and progression of breast cancer.^36^ Su et al reported that snoRNAs (SNORD15A, SNORD15B, SNORD22, SNORD17, and SNORD87) and fibrillarin (an enzyme nucleolar nucleoprotein, snoRNP) were frequently overexpressed in mouse and human breast cancer as well as prostate cancer and proved their correlation with tumorigenicity, revealing the important role of snoRNA in the regulation of cancer biogenesis.^37^ Meanwhile, the expression of SNORD46 and SNORD89 was confirmed to significantly decrease in breast cancer tissues.^38^ Deletion of SNORD3A or SNORD118 in human breast cancer cells resulted in a p53-dependent antitumor stress response.^39^ The invasion of SNORD3A knockout tumor cells significantly decreased and the tumorigenicity of cancer cells completely disappeared when SNORD118 was completely knocked out. Furthermore, the p53-dependent antitumor nucleolar surveillance pathway was activated when SNORD3A or SNORD118 was deleted, suggesting the mechanism by which SNORD3A and SNORD118 regulate tumor occurrence and development.^39^ The expression of SNORD50A decreased in breast cancer tissues, and the prognosis of patients with higher expression of SNORD50A was found to be better.^40^ SNORD50A delayed the proliferation of breast cancer cells by inhibiting the expression of mitotic-related genes, indicating that SNORD50A may work as a breast cancer tumor suppressor.^40^ Brain metastasis is a serious complication of breast cancer. The differential expression of snoRNAs in brain metastatic and nonmetastatic breast cancer tissues was identified. The results showed that SNORA71B was overexpressed in breast cancer cells associated with brain metastasis; significantly promoted the proliferation, migration, and invasion of breast cancer cells; and induced the EMT of metastatic breast cancer cells in the brain.^41^ The expression of SNORA71A, which is related to RMT, in metastatic breast cancer tissues was found to be enhanced compared with that in nonmetastatic breast cancer samples.^42^ In addition, SNORA71A promoted proliferation, migration, invasion, and EMT. Researchers used gene set enrichment analysis (GSEA), found that SNORA71A up-regulated Rock2 in the TGF-β signaling pathway, and proved that SNORA71A increased the half-life of Rock2 mRNA by binding to the mRNA stability-regulating protein G3BP1.^42^

### SnoRNAs in leukemia

In 2012, Teittinen et al used large-scale parallel sequencing (SOLiD) technology to analyze the expression of snoRNAs in different leukemia cell lines and verified the expression profile.^43^ The differentially expressed genes in acute myeloid leukemia (AML), pre-B-ALL, T-ALL, and other leukemic cell subsets were preliminarily screened. For example, the comparison between T-ALL and pre-B-ALL revealed 46 differentially expressed snoRNAs. Subsequently, the expression of nine snoRNAs was detected, among which four snoRNAs (SNORD49A, SNORD55, SNORD105, and SNORD110) were verified.^43^ In the same year, Valleron et al reported negative changes in SNORD112–114 at the DLK1-DIO3 locus in APL. Further experiments in ATRA-treated APL patients showed that ATRA directly targeted PML-RARAα and led to granulocyte differentiation and that the expression of the SNORD112-114 gene cluster in the DLK1-DIO3 site decreased during the process.^44^ These results were supported and strengthened by the research of Liuksiala et al who showed that the DLK1-DIO3 locus and SNORD112-114 in this locus were related to leukemia and stem cell pluripotency.^45^ Ronchetti et al clarified the expression of snoRNA in CLL and different leukemic cell subsets. First, array analysis showed no significant difference between the expression profile of snoRNA in CLL cells and normal memory cells, immature cells, and marginal zone B cells, but a few down-regulated transcripts could be found. In addition, the expression of SNORA70F was significantly down-regulated in CLL with poor prognosis, which may be related to the down-regulation of its host gene COBLL1. Finally, the research team established an independent model to evaluate the prognosis of CLL. Its principle is based on the expression of SNORA74A and SNORD116-18. These results identified the CLL-related biomarkers to predict the clinical outcome of early CLL.^46^ In 2017, Zhou et al found that the formation of Box C/D snoRNA/RNP and rRNA 2′-O-methylation were necessary for the colony formation ability of leukemia cells *in vitro* and leukemia cell viability *in vivo*. For example, the deletion of SNORD34, SNORD35A, SNORD43, and SNORD104 led to disordered rRNA methylation and a decrease in cell volume and the repair speed of damaged proteins. Finally, they determined that AML1 ETO-mediated leukemia was often induced by AES to form Box C/D snoRNA/RNP. In this process, snoRNA is a common downstream target regulated by various oncogenes and related to the self-renewal of leukemia stem cells.^47^

Warner et al optimized the methods of library preparation and bioinformatics analysis to determine and quantify the expression of snoRNAs in AML and normal hematopoietic cell populations. Similar to previous studies, the expression of snoRNA at the DLK-DIO3 site was the highest in CD34^+^ cells and decreased rapidly due to granulocyte differentiation. A more novel result showed similar snoRNAs at the snorf/SNRPN site but with different expression patterns. Eighty-two paternally expressed snoRNAs included at the snorf/SNRPN site were highly expressed in CD34^+^ cells; however, their expression in B and T cells could not decrease rapidly due to granulocyte differentiation, suggesting that snoRNA was specifically expressed in the development and lineage of human hematopoiesis.^48^ Based on the comparison of the expression of SNORD42 in leukemia cells, CD34^+^ progenitor cells, monocytes, and granulocytes in patients with primary AML, the expression of SNORD42 in leukemia cells was significantly enhanced. By knocking out the SNORD42A gene *in vitro*, the colony formation was weakened and cell proliferation was inhibited. In addition, the deletion of SNORD42A, which acted as a Box C/D snoRNA, decreased 2′-O-methylation at uridine 116 of 18S ribosomal RNA which was associated with a specific decrease in the translation of ribosomal proteins and a reduced volume of leukemia cells.^49^

### SnoRNAs in lung cancer

Non-small-cell lung cancer (NSCLC) ranks as the top cancer killer in the population. Early detection of NSCLC helps improve the prognosis, and new evidence showed that snoRNAs are associated with the occurrence and prognosis of NSCLC. Liao et al aimed to identify snoRNAs that could act as biomarkers of early-stage NSCLC.^50^ SnoRNA characterization was performed in NSCLC tissues and adjacent normal lung tissues, and six snoRNAs were identified to be overexpressed in lung cancer tissues. Among them, the expression of SNORD33, SNORD66, and SNORD76 in the plasma of NSCLC patients was significantly enhanced compared with that in healthy individuals. SNORD33 exhibited 81.1% sensitivity and 95.8% specificity in distinguishing NSCLC, healthy subjects, and COPD patients.^50^ SNORA42 was also proven to be an oncogene in lung cancer.^51^ The tumorigenicity of NSCLC cell lines was down-regulated when SNORA42 was reduced, whereas the cell growth and colony formation ability of human bronchial epithelial cell lines were up-regulated when SNORA42 was improved.^51^ Therefore, SNORA42 is thought to be an oncogene in lung cancer, as also verified by other researchers.^51^ Mannoor et al analyzed the differential expression of snoRNAs in tumor-initiating cells (TICs) of NSCLC, showing that the expression of SNORA3 and SNORA42 was negatively correlated with the survival rate of NSCLC patients.^52^ In this study, the expression of SNORA42 in CD133^+^ cells was significantly higher than that in CD133^−^ cells. SNORA42 knockout significantly reduced the proliferation of TICs *in vitro*. The expression of SNORA42 was related to the expression of stem cell core transcription factors in TICs.^52^ In 2014, Gao et al described snoRNA maps of 12 NSCLC tissues.^53^ They identified six snoRNAs related to the overall survival rate of NSCLC and used SNORA47, SNORA68, and SNORA78 to establish a model to predict the overall survival rate of NSCLC.^53^ In 2015, Su et al in the same team explored a new and more sensitive noninvasive diagnostic method for lung cancer by detecting snoRNA in human sputum samples.^54^ An analysis of sputum samples from 59 patients with lung cancer and 61 healthy smokers showed that the sensitivity of snoRNA in sputum was 74.58% for the diagnosis of lung cancer, significantly higher than that of sputum cytology (45.76%), and the specificity of snoRNA was 83.61%.^53^^,^^54^ The expression of the snoRNP core protein NOP10 significantly increased in NSCLC and was negatively correlated with the prognosis of NSCLC patients.^55^ NOP10 knockdown led to a decrease in Box H/ACA snoRNA pseudouridylation, thereby inhibiting the proliferation and migration of tumor cells. Meanwhile, the expression of SNORA65, SNORA7A, and SNORA7B increased in NSCLC, proving that NOP10 may be a treatment target and diagnostic biomarker of NSCLC.^55^

### SnoRNAs in prostate cancer

At present, prostate cancer (PCa) remains the second leading cause of cancer-related death among males worldwide. The overall expression of snoRNA differed more than that of the miRNA in different stages of PCa.^56^ In a subsequent analysis, the expression of SNORD44, SNORD78, SNORD74, and SNORD81 significantly increased in PCa.^56^ In the same year, Francesco et al performed RNA sequencing from PCa specimens and screened 21 differentially expressed snoRNAs in metastatic and non-metastatic PCa.^57^ Among them, SNORA55 could be repeatedly detected in the serum samples of PCa patients. The up-regulation of SNORA55 could predict the progression of PCa, and as verified in PCa cell lines, the silencing of SNORA55 could significantly inhibit cell proliferation and migration.^57^ SNORA42 was confirmed to be significantly up-regulated in PCa tissues, and its expression was three times that in adjacent normal tissues. This finding was positively correlated with the progression of PCa. GO and KEGG analyses showed that SNORA42-regulated downstream proteins were involved in cell development, adhesion, and differentiation, and they may be involved in the cGMP-PKG signal transduction pathway.^58^

### SnoRNA in gliomas

Gliomas originate from glial cells in the brain or spine, accounting for 30% of all brain and central nervous system tumors and 80% of all malignant brain tumors. Bin et al found that the expression of SNORD47 was down-regulated in glioma tissue and negatively correlated with the stage of glioma.^59^ The expression of SNORD47 was positively correlated with survival time in glioma patients. SNORD47 inhibited the proliferation of glioma cells and induced G2 arrest. The invasion and EMT of glioma cells were reduced after up-regulating SNORD47. Thus, SNORD47 produced an inhibitory effect on glioma, thus suggesting a new approach for the treatment of glioma.^59^ Similarly, Xian et al found that the expression of SNORD44 in glioma was significantly down-regulated, and SNORD44 overexpression suppressed the expression of MMP2, MMP9, and the proliferation marker Ki67, thus inhibiting the growth, invasion, and migration of glioma.^60^ SNORD76 was produced by selective splicing of the third intron of GAS5.^61^ Overexpression of SNORD76 effectively inhibited the proliferation of glioma cells, whereas inhibiting the expression of SNORD76 significantly promoted the proliferation of glioma cells. SNORD76 limits the cell cycle of tumor cells to the S phase by regulating the expression and phosphorylation of Rb. Analysis of clinical glioma samples showed that the expression of SNORD76 had a significant correlation with WHO grade; however, no significant correlation was observed between GAS5 and WHO grade.^61^ Pediatric high-grade gliomas (pHGGs) are fast-growing and fatal primary malignant brain tumors derived from glial stem cells or progenitor cells with high heterogeneity.^62^ PHGGs are remarkably different from human gliomas, but they have highly invasive clinical behavior similar to that of adult gliomas. The expression of 36 snoRNAs in the HBII-52 snoRNA cluster in pHGG tissue was reported to be significantly reduced. Meanwhile, h3f3a and TP53 mutations led to significant changes in the expression of snoRNA in pHGG tissue.^63^

### SnoRNA in other cancers

Little research is available on the mechanism of snoRNAs in clear cell renal cell carcinoma (ccRCC). Shang et al found that the expression of SNORD63 and SNORD96A in ccRCC increased significantly by comparing 516 ccRCC patients with 71 healthy controls in the SNORic and TCGA databases.^64^ SNORD63 in the urinary system and SNORD96A in plasma could be potential non-invasive diagnostic biomarkers.^64^

Gastric cancer (GC) is a malignant tumor originating from the gastric mucosal epithelium.^65^^,^^66^ A study revealed that the expression of SNORD105B increased in the tissues and peripheral blood of GC patients. Overexpression of SNORD105B significantly promoted the proliferation, migration, and invasion of GC cells. SNORD105B promoted the progression of GC cells by regulating the c-MYC signaling pathway in combination with ALDOA.^67^ Analysis of 79 GC tissues and adjacent tissues revealed that SNORA21 expression was significantly increased in GC tissues and GC cell lines.^68^ The expression of SNORA21 was closely related to distant metastasis and lymphatic metastasis of GC. Thus, SNORA21 is also an important indicator of poor prognosis in GC patients.^68^

In addition, differentially expressed snoRNAs could be found in gallbladder cancer (GBC). Qin et al performed microarray analysis between GBC and adjacent normal tissue and found that SNORA21 was the most down-regulated snoRNA in GBC tissue.^69^ Overexpression of SNORA21 inhibited the proliferation, migration, and invasion of GBC cells. Therefore, overexpression of SNORA21 could significantly inhibit the growth of GBC *in vivo*, and SNORA21 may act as a potential new treatment target for GBC.^69^

The expression of SNORA23 was correlated with tumor invasion grade and survival time of patients with pancreatic ductal adenocarcinoma (PDAC). Lin et al detected the expression of snoRNA in PDAC cell lines and found that the expression of SNORA23 in highly metastatic MIA PaCa2 or Suit2-HLMC cells was higher than that in parental cells.^70^ SNORA23 overexpression increased the invasiveness and colony formation of PDAC cells. Tumor growth, tumor cell dissemination, and liver metastasis were inhibited after injection of antisense oligonucleotides against SNORA23 into xenogeneic tumor-transplanted nude mice.^70^

Head and neck squamous cell carcinoma (HNSCC) is a common malignant tumor with high mortality and poor prognosis due to the lack of predictive biomarkers and effective treatment. Lu et al screened snoRNAs related to the prognosis of HNSCC by Cox regression analysis and explored their function by co-expression analysis and GSEA.^71^ Finally, five snoRNAs (SNORD114-17, SNORA36B, SNORD3A, SNORD3F, and SNORD78) associated with the prognosis of HNSCC were identified, with high sensitivity and specificity, and they were involved in regulating the phenotype of malignant tumors and DNA/RNA editing.^71^

Ovarian cancer (OV) is the main cause of gynecological cancer-related death. Zhu et al screened out snoRNAs related to the prognosis of OV patients by analyzing the data of 379 OV patients in the TCGA database and the differentially expressed snoRNAs in OV spherical cells and ovarian cells. Finally, SNORD89 was selected as the candidate for use in further analysis.^72^ SNORD89 was highly expressed in OV stem cells and associated with poor prognosis in OV patients. The overexpressed SNORD89 led to an increase in stem cell markers, the proportion of cells in the S-phase cell cycle, and the proliferation, invasion, and migration of ovarian cells and OV cells. By contrast, these phenomena were reversed after SNORD89 was knocked out. In addition, up-regulation of mRNA and protein and the expression of c-Myc and Notch1 were found downstream of SNORD89. In summary, SNORD89 may act as an oncogene in ovarian tumors by promoting cell stemness by regulating the Notch1-c-Myc pathway, thus leading to poor prognosis in OV patients.^72^

By analyzing the RNA sequencing data of 257 patients with sarcomas in the TCGA database, four diagnostic snoRNAs (SNORD3A, SNORA73B, SNORD46, and SNORA26) were identified.^73^ The p53 gain-of-function mutation is an important cause of sarcoma metastasis. Studies have revealed that p53 mutation could lead to a high expression of a series of snoRNAs in osteosarcoma cells, including SNORA7A and SNORD8D.^74^ Doxorubicin is a commonly used drug for the treatment of osteosarcoma, but doxorubicin resistance often leads to insensitivity to osteosarcoma treatment. Therefore, identifying the relevant mechanism of doxorubicin resistance is necessary. Screening showed that SNORD3A, SNORA13, and SNORA28 were closely related to doxorubicin resistance in osteosarcoma.^75^

Significant changes in the snoRNA expression in tumor cells and tissues and body fluids have important biological and diagnostic implications. These significantly altered snoRNAs provide new biomarkers for the diagnosis of tumors as well as new targets for tumor therapy (Table 1).

**Table 1:** The functions of snoRNAs in cancers.

snoRNA name	Cancer type	Expression	Mechanism, pathway, or target	Functions	Reference
SNORA 18L5	Hepatocellular carcinoma	Up-regulation	p53	Increase ribosome biogenesis, facilitate ribosomal RNA maturation, and alter the localization of RPL5 and RPL11, allowing for increased MDM2-mediated proteolysis of p53 and cell cycle arrest	^15^
SNORA15	Colorectal carcinoma	Up-regulation	Unknown	Unproved	^23^
SNORA21	Colorectal carcinoma	Up-regulation	Unknown	Be involved in epithelial cell differentiation, morphogenesis, and cell adhesion	^21^
	Gallbladder cancer	Down-regulation	Ecadherin, N-cadherin, vimentin, c-Myc and Cyclin D1	Inhibit GBC cell migration and invasion by down-regulating the EMT process of GBC cells	^69^
	Gastric cancer	Up-regulation	Unknown	Be associated with distant metastasis and lymph node metastasis in GC patients	^68^
	Lung cancer	Up-regulation	Unknown	Unproved	^52^^,^^53^
SNORA23	Colorectal carcinoma	Up-regulation	Unknown	Unproved	^24^
	Pancreatic ductal adenocarcinoma	Up-regulation	SYNE2	Promote PDAC cell survival and invasion, and growth and metastasis of xenograft tumors through modulation of ribosome biogenesis	^70^
SNORA24	Colorectal carcinoma	Up-regulation	Unknown	Unproved	^24^
SNORA26	Sarcomas	Up-regulation	Rap1, Hippo, MAPK, sphingolipid, TGF-β, Wnt	Enrich in cell differentiation, regulation of cell development, regulation of transcription, cell–cell signaling, and activation of protein kinase B activity	^73^
SNORA3	Lung cancer	Up-regulation	Unknown	Unproved	^52^
SNORA31	Leukemia	Down-regulation	Unknown	Unproved	^46^
SNORA36B	Head and neck squamous cell carcinoma	Down-regulation	Unknown	Unproved	^71^
SNORA41	Colorectal carcinoma	Up-regulation	Unknown	Unproved	^23^
SNORA42	Colorectal carcinoma	Up-regulation	Unknown	Increase cell proliferation, tumorigenicity, migration, invasion, and anoikis resistance in colon cancer cells	^20^
	Lung cancer	Up-regulation	Unknown	Be associated with the expression of stem cell-core transcription factors in lung tumor-initiating cells	^51^
	Prostate cancer	Up-regulation	Metabolic pathways	Enhance prostate cancer cell viability, migration, and EMT	^58^
SNORA47	Lung cancer	Up-regulation	Unknown	Unproved	^53^
SNORA52	Hepatocellular carcinoma	Down-regulation	Unknown	Function as a potential diagnostic and prognostic biomarker for HCC patients	^132^
SNORA55	Prostate cancer	Up-regulation	Unknown	Be involved in the cell-to-cell signaling and interaction	^57^
SNORA6	Leukemia	Down-regulation	Unknown	Unproved	^46^
SNORA62	Leukemia	Down-regulation	Unknown	Unproved	^46^
SNORA65	Lung cancer	Up-regulation	Unknown	Unproved	^55^
SNORA68	Lung cancer	Up-regulation	Unknown	Unproved	^53^
SNORA70F	Leukemia	Down-regulation	Unknown	Unproved	^46^
SNORA71A	Breast cancer	Up-regulation	ROCK2, TGF-β	Promote breast tumor growth and enhance metastasis of breast cancer	^42^
	colorectal carcinoma	Up-regulation	LBP, NF-kappa B, Toll-like receptor	Promote CRC cell migration and invasion	^24^
SNORA71B	Breast cancer	Up-regulation	EMT	Promote the proliferation, migration, and invasion of breast cancer cells with different metastatic abilities	^41^
SNORA71C	Leukemia	Down-regulation	Unknown	Unproved	^46^
SNORA73B	Sarcomas	Up-regulation	Oxidative phosphorylation, metabolic pathways, NF-kappaB, stimulatory C-type lectin receptor	Enrich in cell cycle, regulation of protein ubiquitination, and RNA metabolic process	^73^
SNORA74A	Leukemia	Up-regulation	Unknown	Unproved	^46^
SNORA78	Lung cancer	Up-regulation	Unknown	Unproved	^53^
SNORA7A	Lung cancer	Up-regulation	Unknown	Unproved	^55^
SNORA7B	Lung cancer	Up-regulation	Unknown	Unproved	^55^
SNORD104	Leukemia	Up-regulation	Unknown	Lead to the disorder of rRNA methylation, and also lead to the decrease of cell volume and the repair speed of damaged protein	^47^
SNORD105	Leukemia	Up-regulation	Unknown	Unproved	^43^
SNORD105B	Gastric cancer	Up-regulation	ALDOA/C-Myc Pathway	Be associated with tumor size, differentiation, and pathological stage in GC as well as affect proliferation, migration, and invasion in multiple GC cell lines	^67^
SNORD110	Leukemia	Down-regulation	Unknown	Unproved	^43^
SNORD112–114	Leukemia	Up-regulation	Unknown	Unproved	^45^
SNORD113-1	Hepatocellular carcinoma	Down-regulation	MAPK/ERK, TGF-β	Function as a tumor suppressor role in HCC and be important as a potential diagnostic and therapeutic target for HCC	^10^
SNORD114-17	Head and neck squamous cell carcinoma	Up-regulation	PI3K/AKT, the ECM receptor	Be involved in the regulation of cell adhesion, invasion, and metastasis	^71^
SNORD116-18	Leukemia	Up-regulation	Unknown	Unproved	^46^
SNORD126	Hepatocellular carcinoma	Up-regulation	FGFR2, PI3K-AKT	Promote the progression of HCC	^11^,^12^
SNORD12B	Colorectal carcinoma	Up-regulation	Unknown	Unproved	^19^
SNORD12C/SNORD106	Colorectal carcinoma	Up-regulation	Unknown	Unproved	^26^
SNORD14E	Colorectal carcinoma	Up-regulation	Unknown	Be involved in systemic lupus erythematosus, alcoholism, viral carcinogenesis, transcriptional mis-regulation in cancer	^26^
SNORD15A	Breast cancer	Up-regulation	p53	Compromise tumorigenicity both *in vitro* and *in vivo* via activation of p53	^37^
SNORD15B	Breast cancer	Up-regulation	p53	Compromise tumorigenicity both *in vitro* and *in vivo* via activation of p53	^37^
SNORD17	Colorectal carcinoma	Up-regulation	Unknown	Unproved	^26^
	Hepatocellular carcinoma	Up-regulation	p53	Promote the growth and tumorigenicity of HCC cells *in vitro* and *in vivo*	^16^
SNORD1C	Colorectal carcinoma	Up-regulation	Unknown	Involvement of snoRNAs in the regulation of ribosomes, rRNA processing, RNA splicing, and translation regulation	^25^
SNORD22	Breast cancer	Up-regulation	p53	Compromise tumorigenicity both *in vitro* and *in vivo* via activation of p53	^37^
SNORD28	Lung cancer	Up-regulation	Unknown	Unproved	^53^
SNORD33	Colorectal carcinoma	Down-regulation	Unknown	Unproved	^23^
	Lung cancer	Up-regulation	Unknown	Unproved	^50^
SNORD34	Leukemia	Up-regulation	Unknown	Lead to the disorder of rRNA methylation, and also lead to the decrease of cell volume and the repair speed of damaged protein	^47^
SNORD35A	Leukemia	Up-regulation	Unknown	Lead to the disorder of rRNA methylation, and also lead to the decrease of cell volume and the repair speed of damaged protein	^47^
SNORD3A	Head and neck squamous cell carcinoma	Down-regulation	Unknown	Unproved	^71^
SNORD3F	Head and neck squamous cell carcinoma	Up-regulation	Unknown	Be related to RNA editing	^71^
SNORD42A	Leukemia	Up-regulation	2′-O-methylation at Uridine 116 of 18S rRNA	An important snoRNA for the proliferation of leukemic cells	^49^
SNORD43	Leukemia	Up-regulation	Unknown	Lead to the disorder of rRNA methylation, and also lead to the decrease of cell volume and the repair speed of damaged protein	^47^
SNORD44	Colorectal carcinoma	Down-regulation	Caspase-dependent pathway, PI3K/Akt	Inhibit CRC cell proliferation and induced caspase-dependent cell apoptosis	^22^
	Gliomas	Down-regulation	Caspase-dependent apoptosis pathway	Facilitate the apoptosis with the inhibited proliferation, invasion, and migration of glioma cells	^60^
	Prostate cancer	Up-regulation	Unknown	Unproved	^56^
SNORD46	Breast cancer	Downregulation	Unknown	Unproved	^38^
	Sarcomas	Up-regulation	Unknown	Enrich in the regulation of gene expression, DNA replication, DNA repair, cell division, cell cycle, cell proliferation, oxidative phosphorylation, and response to drug	^73^
SNORD47	Gliomas	Down-regulation	Unknown	Inhibit glioma cell growth, proliferation, colony formation, invasion, and migration, and induces G2-phase arrest *in vivo* and *in vitro*.	^59^
SNORD49A	Leukemia	Up-regulation	Unknown	Unproved	^43^
SNORD50	Colorectal carcinoma	Down-regulation	Methylation of C2848 in 28S rRNA	Unproved	^32^
SNORD50(A/B)	Breast cancer	Down-regulation	k-Ras	Downregulate mitosis-related genes, prolonged mitosis, repressed colony-forming ability, and clinical analyses	^36^
SNORD52	Hepatocellular carcinoma	Up-regulation	CDK1	Be associated with the poor prognosis of patients with HCC, acting as a functionally relevant snoRNA in HCC	^17^
SNORD55	Leukemia	Up-regulation	Unknown	Unproved	^43^
SNORD57	Colorectal carcinoma	Up-regulation	Unknown	Unproved	^33^
SNORD63	Clear cell renal cell carcinoma	Up-regulation	Unknown	Unproved	^64^
SNORD66	Lung cancer	Up-regulation	Unknown	Unproved	^50^,^53^
SNORD67	Colorectal carcinoma	Up-regulation	Unknown	Be involved in systemic lupus erythematosus, alcoholism, viral carcinogenesis, transcriptional mis-regulation in cancer	^26^
SNORD73B	Lung cancer	Up-regulation	Unknown	Unproved	^50^
SNORD74	Prostate cancer	Up-regulation	Unknown	Unproved	^56^
SNORD76	Colorectal carcinoma	Up-regulation	Unknown	Unproved	^20^
	Gliomas	Down-regulation	pRb	Arrest glioma cells at the S phase of the cell cycle, which in turn may affect the expression of cell cycle-associated proteins	^61^
	Hepatocellular carcinoma	Up-regulation	Wnt/β -catenin	Suppress cell proliferation by inducing G0/G1 cell cycle arrest and apoptosis	^14^
	Lung cancer	Up-regulation	Unknown	Unproved	^50^
SNORD78	Colorectal carcinoma	Up-regulation	Unknown	Unproved	^20^
	Head and neck squamous cell carcinoma	Up-regulation	Unknown	Unproved	^71^
	Lung cancer	Up-regulation	Unknown	Unproved	^50^
	Prostate cancer	Up-regulation	Unknown	Unproved	^56^
SNORD81	Prostate cancer	Up-regulation	Unknown	Unproved	^56^
SNORD87	Breast cancer	Up-regulation	p53	Compromise tumorigenicity both *in vitro* and *in vivo* via activation of p53	^37^
SNORD89	Breast cancer	Down-regulation	Unknown	Unproved	^38^
	Ovarian cancer	Up-regulation	Notch1-c-Myc	Result in increased stemness markers, S phase cell cycle, cell proliferation, invasion, and migration ability in OV and CA cells	^72^
SNORD96A	Clear cell renal cell carcinoma	Up-regulation	Unknown	Unproved	^64^
SNORD3A	Breast cancer	Up-regulation	p53	Trigger a remarkably potent p53-dependent anti-tumor stress response involving the ribosomal proteins uL5 (RPL11) and uL18 (RPL5)	^39^
	Sarcomas	Down-regulation	Unknown	Enrich in oxidative phosphorylation, regulation of protein ubiquitination, cellular metabolic process, and RNA metabolic process	^73^
SNORD118SCARNA9L	Breast cancer	Up-regulation	p53	Trigger a remarkably potent p53-dependent anti-tumor stress response involving the ribosomal proteins uL5 (RPL11) and uL18 (RPL5)	^39^
	Hepatocellular carcinoma	Up-regulation	Wnt/β-catenin	Inhibit the proliferation of HCC cells by inducing G0/G1 arrest and apoptosis	^13^
SCARNA6	Leukemia	Up-regulation	Unknown	Unproved	^43^
SCARNA22	Colorectal carcinoma	Up-regulation	Unknown	Unproved	^20^
SCARNA15	Colorectal carcinoma	Up-regulation	Unknown	Unproved	^24^

## SnoRNA in genetic disease

### SnoRNA in PWS

PWS is a rare genetic disease that is recognized as a complex neurodevelopmental disorder.^76^ The clinical entity was described for the first time by Prader, Labhart, and Willi in 1956, and it was called Prader–Labhart–Willi syndrome.^77^ In 2014, Bieth et al reported a female patient with PWS who had a paternal deletion in the SNORD116 gene cluster. In this case, the shortest SNORD116 deletion fragment was reported at that time, with only a 118-kb deletion, which was regarded as a highly restricted fragment deletion, and SNORD116 was strongly proven to play an important role in the pathogenesis of PWS.^78^ In 2020, Tan et al reported a 17-year-old PWS patient and found a heterozygous deletion with a length of 71 kb at chr15:25296613–25367633 of the genome constructing hg19. This deletion could not affect the SNURF-SNRPN locus but could result in the loss of some PWS-related noncoding RNAs, including SNORD116. This rare case demonstrated that paternal copy loss of SNORD116 determined most clinical features of PWS.^79^

The loss of function of one or more imprinted paternally expressed genes on the proximal long arm of chromosome 15 has been confirmed as the cause of PWS, and PWS occurs as a result of the loss of function of several paternally expressed genes.^80^^,^^81^ The locus is composed of several paternally expressed protein-coding genes, a piRNA gene cluster, and six different Box C/D snoRNA family members. Among them, SNORD116 (HBII-85) and SNORD115 (HBII-52) are the largest tandem repeat clusters containing 29 and 48 gene copies, respectively. Other snoRNAs exist in the form of single copies [SNORD64 (HBII-13), SNORD107 (HBII-436), and SNORD108 (HBII-437)] or double copies [SNORD109A/B (HBII-438A/B)]^4^^,^^82^, ^83^, ^84^.

SNORD116-deficient mice lost weight more slowly with reduced calorie intake.^85^ Selective disruption of SNORD116 expression in the middle lobe of the hypothalamus could lead to overeating in mice.^86^ Mechanistically, the deletion of SNORD116 up-regulated the mRNA expression of NPY and POMC in the arcuate nucleus of mice.^87^, ^88^, ^89^, ^90^ A study showed that SNORD116 was increased in the weaning and youth periods but not in the neonatal period.^91^ In further experiments, the survival rate of SNORD116 del newborns was significantly improved by taking the GHSR agonist HM01 every day for 2 weeks. In conclusion, GHSR agonists have the potential to reduce PWS mortality.^92^ In the SNORD116^−/−^ model mice, task-dependent changes were found in motor- and anxiety-related behavior, which did not show sex specificity.^93^, ^94^, ^95^ The sleep-wake disturbance is often seen in PWS patients. Studies have suggested that paternally expressed SNORD116 may be a candidate gene inducing PWS patients to easily enter the awake state from rapid eye movement sleep.^96^, ^97^, ^98^, ^99^

SNORD115 has sequence complementarity with the alternative splicing exon VB in the 5-HT receptor, so it could bind the silencing element in VB and regulate the alternative splicing of the 5-HT receptor.^100^^,^^101^ SNORD115 generated a new short RNA, and it was suggested to be classified into the so-called psnoRNA family.^100^, ^101^, ^102^ Cruvinel et al found a complex composed of ZNF274 and the H3K9 methyltransferase SETDB1, which binds to the silent maternal SNORD116 to protect the imprinted central DNA of PWS from demethylation.^103^ ZNF274 was knocked out in the PWS imprinting center, the silenced maternal allele expression in the neurons of the PWS IPSC strain was saved and DNA methylation was not affected, indicating that the ZNF274 complex is a separate imprinting marker.^104^^,^^105^ A genome-wide array analysis was performed after up-regulating SNORD115 and SNORD116 in HEK 293T cells to identify the downstream targets of SNORD115 and SNORD116. The results showed that up-regulation of SNORD116 changed the expression of more than 200 genes, most of which were mRNAs. The up-regulation of SNORD115 also affected the expression of SNORD116.^106^

### SnoRNA in Labrune syndrome

Labrune syndrome, as a neurological disease, is essentially identified as leukoencephalopathy with calcifications and cysts (LCC). As early as 1996, P. Labrune et al reported for the first time the existence of Labrune syndrome in three unrelated children, and they found cognitive decline and spastic seizures accompanied by extrapyramidal and cerebellar symptoms.^107^ Emma et al collected clinical data and biological samples of 40 LCC patients over a 12-year period and confirmed a biallelic mutation in the SNORD118 fragment of chromosome 17, which may be the cause of LCC, by Sanger sequencing.^108^ Similarly, Anan et al reported a 12-year-old boy with characteristic LCC features in 2019, and sequencing analysis revealed a mutation in SNORD118 on the double allele.^109^ In 2020, Yanick et al collected information on LCC patients from 56 families and 64 individuals for cohort observation and analysis. The results showed 44 mutation types of SNORD118 that may be the cause of disease, and genetic correlation of LCC was only reported in three of the 56 families.^110^

### SnoRNA in myelodysplastic syndromes

Myelodysplastic syndromes (MDSs) are a group of heterogeneous myeloid clonal diseases originating from hematopoietic stem cells. They are characterized by abnormal differentiation and development of myeloid cells, manifested as ineffective hematopoiesis, refractory hemocytopenia, hematopoietic failure, and high-risk transformation to AML.^111^ DDX41 mutation is an important factor leading to MDS, and frameshift mutations at positions D52 and D140 of aspartic acid could lead to the inactivation of the DDX41 protein.^112^, ^113^, ^114^, ^115^, ^116^ DDX41, which belongs to the DEAD box protein family and is characterized by the conserved motif Asp-Glu-Ala-Asp (DEAD), is a putative RNA helicase. It is implicated in a number of cellular processes involving alteration of RNA secondary structure, such as translation initiation, nuclear and mitochondrial splicing, and ribosome and spliceosome assembly. Mutation or deletion of DDX41 could lead to abnormal expression of snoRNA, which could result in abnormal ribosomal assembly and protein synthesis. SnoRNAs have been proven to play an important regulatory role in the occurrence of MDS.^112^

### SnoRNA in immunity

Systemic lupus erythematosus (SLE) is an autoimmune disease in which the body's immune system mistakenly attacks many healthy tissues. Lai et al analyzed the T-cell RNA transcriptome expression profiles of three SLE patients and three normal controls through a microarray and preliminarily screened out 18 differentially expressed ncRNAs in T cells of SLE patients. These ncRNAs were subsequently verified in T cells from 23 SLE patients and 17 normal controls. The results showed that the expression of SNORA12 was significantly reduced in T cells of SLE patients. The plasmid encoding SNORA12 was transfected into Jurkat cells and changes in gene expression were detected. The expression levels of two and 15 genes increased and decreased, respectively, and they were significantly related to the SLE pathway in the KEGG and genome maps. In conclusion, overexpression of SNORA12 changed the expression of CD69, decreased the expression of HIST1H4K, and inhibited the secretion of interferon γ, thus participating in the immune pathogenesis of SLE.^117^

Herpes simplex encephalitis (HSE) refers to encephalitis caused by the herpes simplex virus, 90% of cases of which are caused by herpes simplex virus-1 (HSV-1), which is usually sporadic. Fabien et al reported five HSE patients unrelated to environmental infection, and each patient was heterozygous for the SNORA31 mutation. By knocking out SNORA31 on double or single alleles, they found that the deletion of SNORA31 led to the increased susceptibility of human pluripotent stem cell-derived cortical neurons to HSV-1. Subsequently, transcriptome analysis of SNORA31 mutant neurons showed an abnormal response to HSV-1 stimulation. Therefore, SNORA31 may mediate the innate immunity of the central nervous system neurons to HSV-1 through a unique mechanism.^118^

## SnoRNA in inflammation

Osteoarthritis (OA) is a type of degenerative arthritis caused by the destruction of articular cartilage and bone. The diagnosis and treatment of OA are still hampered by the lack of biomarkers to evaluate disease progression. Mandy et al attempted to find biomarkers for OA by analyzing the expression pattern of snoRNAs in OA. First, they identified six differentially expressed snoRNAs (SNORD113, SNORA3, SNORD88, SNORA73, and SNORD38) in young and aging joints, and in animal experiments, SNORD88 and SNORD38 changed significantly in the process of joint aging, suggesting their potential value in the diagnosis of OA.^119^ Similarly, Mandy et al screened differentially expressed snoRNAs in articular cartilage from young, elderly, and OA knee joints by microarray analysis; they found that the expression of SNORD96A and SNORD44 was related to the aging of articular cartilage and that of SNORD26 and SNORD116 was related to OA.^120^ Overexpression of SNORD26 and SNORD96A led to changes in chondrogenesis, hypertrophy, rRNA, and OA-related gene expression.^120^ In addition, Ellen et al found that OA synovial fluid could affect the expression of SNORD3A, which was increased by BMP7, resulting in a change in chondrocyte phenotype. The reduced expression of SNORD3A led to a decrease in the rRNA level and translation ability, while the induced expression of SNORD3A was accompanied by an increase in 18S and 28S rRNA and protein translation. These results demonstrated that snoRNAs maintain biological significance in OA and play an essential role in chondrocyte differentiation, rRNA level, and protein translation ability.^121^ COVID-19 can cause a strong inflammatory response, and good biomarkers to predict the degree of inflammation in COVID-19 are lacking. Detection of 29 COVID-19 patients found that the expression of snoRNAs significantly differed among severe, moderate, and asymptomatic patients, and the expression of five snoRNAs was significantly higher in severe and moderate symptomatic patients than in asymptomatic patients.^122^ Macrophages are an important part of the human innate immune system as they have powerful functions in recognizing, phagocytizing, and removing bacteria and foreign bodies. In the initial stage of inflammation, macrophages are induced by different stimulating factors, and most of them are polarized into the M1 type through different pathways, which are involved in the elimination of pathogens. In the recovery stage of inflammation, macrophages transform from the M1 type to the M2 type and clear the apoptotic PMN through the burial pathway. High-throughput sequencing analysis showed that the expression of snoRNA changed significantly in the polarization process of macrophages. Thus, regulating the expression of snoRNA could change the polarization state of macrophages.^123^

## SnoRNA in metabolism

The significant pathological features of metabolic syndrome and type II diabetes are dyslipidemia and corresponding fatty toxicity. Excessive lipids in the body lead to cell dysfunction and induce cell apoptosis through oxidative stress-related mechanisms. Arthur et al found that SNORA73 knockout in cells could antagonize lipid-induced cell death and general oxidative stress and prevent steatosis and lipid-induced oxidative stress and inflammation. This protection against metabolic stress is associated with extensive reprogramming of mammalian rapamycin signal axis target-dependent oxidative metabolism.^124^

Clinical studies have demonstrated that long-term use of the antiretroviral drug tenofovir could cause abnormal bone metabolism and bone loss. Tenofovir could also regulate the function of osteoclasts and the expression of SNORD32A in osteoclasts.^125^

## SnoRNA in senescence

In 1961, Leonard Hayflick found that even given the most appropriate conditions for cell growth, cell failure occurs when cells divide into a certain number of generations, thus causing the cell cycle to enter an “irreversible” stagnation state. On the basis of this phenomenon, Hayflick proposed the concept of cellular senescence for the first time.^126^^,^^127^ B cells are important cells in the adaptive immune system. When the body is infected by a virus or becomes vaccinated, B cells produce corresponding antibodies. However, the aging of the body reduces its ability to fight against viral infection or vaccine-related immunity. B-cell aging may be an important reason for this phenomenon. A study on B-cell immune aging revealed that the expression of SNORD123 in B cells of old mice was significantly higher than that of young mice.^128^ With the increase in population aging, the number of patients with age-related osteoporosis has gradually increased. Bone marrow mesenchymal stem cells (BMSCs) could differentiate into osteoblasts and maintain normal bone mass. The aging of BMSCs is an important reason for the occurrence of senile osteoporosis. A replicative aging model of BMSCs *in vitro* showed that snoRNAs changed significantly in the senescence process of BMSCs. Small RNA sequencing showed that 63 snoRNAs had significant differences, and 32 of them were verified.^129^ The topic of prevention and delaying of age-related diseases and increasing the human lifespan has been explored since ancient times. The major discoveries of scientific research make it possible to increase lifespan. A new snoRNA was found in *Drosophila* enterocytes and named snoRNA jouvence.^130^ The study revealed that a mutation of snoRNA jouvence reduced the lifespan of *Drosophila*, whereas overexpression of snoRNA jouvence in enterocytes prolonged it. Mutation of the snoRNA junction led to the loss of pseudouridylation of 18S and 28S rRNA in *Drosophila*.^130^ Due to snoRNA's strong conservation, snoRNA jouvence may prolong the lifespan of other species. The expression of the snoRNA junction was detected in human tumor cells and primary cells.^131^ Overexpression of the snoRNA complex in human cells could promote cell proliferation. By contrast, knocking down the expression of snoRNA jouvence in human cells could inhibit cell proliferation.^131^

## Conclusions

At present, with the development of sequencing technology and microarrays, an increasing number of disease-related snoRNAs have been identified, but the research fields are still mostly limited to tumors and genetics, and research on inflammation and other fields remains to be developed. Regarding the depth of research, the current reports are more limited to the screening of snoRNAs and verification of the relationship between snoRNAs and diseases. The understanding of the mechanism is still incomplete, especially the role of snoRNAs in cell signal transduction pathways. Animal models play an indispensable role in the study of snoRNAs, but the current animal models for snoRNA research are still insufficient. Crisp/cas9 technology regulating gene expression provides favorable support for the study of snoRNAs in animals. Studying the regulatory role of snoRNAs in diseases could help people further understand diseases and develop specific diagnostic and treatment technologies.

## Author contributions

Conceptualization: Xiaodong Chen and Chuandong Wang; editing: Xinhai Zhang, Shujun Xia, Chuandong Wang, Fengbin Yu, Fei Xiao, Chenglong Wang and Jianping Peng.

## Conflict of interests

The authors declare that the research was conducted in the absence of any commercial or financial relationships that could be construed as a potential conflict of interest.

## Funding

This work is supported by the 10.13039/501100001809National Natural Science Foundation of China (No. 82172473, 82072462, 81802191), the 10.13039/501100007129Natural Science Foundation of Shandong Province, China (No. ZR2019PH068), Public Welfare Basic Research Program of Zhejiang Province, China (No. LY20H060002) and 2018 Xinhua-uOttawa joint clinical research, China (No. 18JXO08).
